# Older Cancer Patients’ User Experiences With Web-Based Health Information Tools: A Think-Aloud Study

**DOI:** 10.2196/jmir.5618

**Published:** 2016-07-25

**Authors:** Sifra Bolle, Geke Romijn, Ellen M A Smets, Eugene F Loos, Marleen Kunneman, Julia C M van Weert

**Affiliations:** ^1^ Amsterdam School of Communication Research/ ASCoR Department of Communication Science University of Amsterdam Amsterdam Netherlands; ^2^ Faculty of Behavioural and Movement Sciences Department of Clinical Psychology VU University Amsterdam Netherlands; ^3^ Department of Medical Psychology Amsterdam Medical Center/ AMC University of Amsterdam Amsterdam Netherlands; ^4^ Department of Medical Decision Making Leiden University Medical Center Leiden Netherlands

**Keywords:** user experience, eHealth, usability, think aloud, aging, cancer

## Abstract

**Background:**

Health information is increasingly presented on the Internet. Several Web design guidelines for older Web users have been proposed; however, these guidelines are often not applied in website development. Furthermore, although we know *that* older individuals use the Internet to search for health information, we lack knowledge on *how* they use and evaluate Web-based health information.

**Objective:**

This study evaluates user experiences with existing Web-based health information tools among older (≥ 65 years) cancer patients and survivors and their partners. The aim was to gain insight into usability issues and the perceived usefulness of cancer-related Web-based health information tools.

**Methods:**

We conducted video-recorded think-aloud observations for 7 Web-based health information tools, specifically 3 websites providing cancer-related information, 3 Web-based question prompt lists (QPLs), and 1 values clarification tool, with colorectal cancer patients or survivors (n=15) and their partners (n=8) (median age: 73; interquartile range 70-79). Participants were asked to think aloud while performing search, evaluation, and application tasks using the Web-based health information tools.

**Results:**

Overall, participants perceived Web-based health information tools as highly useful and indicated a willingness to use such tools. However, they experienced problems in terms of usability and perceived usefulness due to difficulties in using navigational elements, shortcomings in the layout, a lack of instructions on how to use the tools, difficulties with comprehensibility, and a large amount of variety in terms of the preferred amount of information. Although participants frequently commented that it was easy for them to find requested information, we observed that the large majority of the participants were not able to find it.

**Conclusions:**

Overall, older cancer patients appreciate and are able to use cancer information websites. However, this study shows the importance of maintaining awareness of age-related problems such as cognitive and functional decline and navigation difficulties with this target group in mind. The results of this study can be used to design usable and useful Web-based health information tools for older (cancer) patients.

## Introduction

An increasing amount of health information is delivered on the Internet [1]. At the same time, more and more patients search the Internet to find information regarding their illness or treatment [2]. This is a fortunate development as the use of Web-based health information tools (eg, Web-based patient education, patient portals, and health-related apps) improves patients’ health-related outcomes [3]. These tools can serve different functions, such as providing information or improving communication with health care providers through the use of so-called “preparatory tools” that support the patient in preparing for consultations and/or in making treatment decisions. Examples of preparatory tools are question prompt lists (QPLs) and decision aids. QPLs are structured lists of questions or topics that patients can use to prepare for a medical encounter by choosing questions they would like to ask their provider during the consultation. QPLs have been found to enhance patient participation and improve emotional and cognitive outcomes in cancer patients [4]. Decision aids are tools that help patients make decisions about their treatment by informing them of treatment options and helping them clarify their values. This helps patients communicate their values and wishes to their health care provider who can use this information to create an optimal treatment plan tailored to the patient [5].

Older patients are an important target group for Web-based health information considering the fact that many diseases (eg, cancer, diabetes, and hypertension) are diseases of older adults [6]. A recent literature review revealed that an increasing number of Web-based health information tools for older patients have been developed and that older patients benefit from the use of these tools as evidenced by improved outcomes such as self-efﬁcacy, blood pressure, hemoglobin levels, and cholesterol levels [7]. These results were especially prevalent for Web-based health information tools with a variety of functions. However, descriptions of development processes are often not published, raising questions about the extent to which these Web-based health information tools are optimally adapted to older patients’ needs and abilities [8]. Hence, we know *that* older individuals use the Internet to search for health information and that this may result in positive health outcomes, but we lack knowledge on *how* they use and evaluate Web-based health information. Older patients experience more difficulties using Web-based technologies compared with younger age groups as they are simply less experienced in using Web-based technologies. Although this problem might resolve itself in the future decades as new generations of older adults have more experience with Web-based technologies, it is to be expected that future older generations will still face usability issues due to age-related problems such as cognitive decline and sensory and functional limitations [9,10]. First, sensory limitations such as visual decline can affect the readability of a website, for example, when small font sizes are used. Second, functional limitations such as the worsening of fine motor skills can cause problems when precise mouse movements are required, for example, using pull-down menus, which only stay open when someone moves over the area with the mouse [11]. Therefore, the use of static navigational elements, such as drop-down menus that stay open until one clicks on a link, has been recommended [9,12]. Third, (age related) cognitive decline can hinder someone’s ability to process information. The more information that is presented on websites, the more difficult it becomes for people with cognitive decline to find required information [12]. For example, Czaja et al [13] demonstrated that the influence of age on the use of technology (ie, computer and Internet use) is mediated by such age-related problems. Involvement of the end user at an early stage in the development process for Web-based health information tools is of high importance to tailor the tools to address such problems [14,15]. Still, literature on user experiences with Web-based health information tools for patients is scarce [16,17]. As a result, cumulative knowledge to be used for the development of Web-based health information tools for older patients is largely missing.

Many studies consider usability, that is, “the extent to which a product can be used by specified users to achieve specified goals with effectiveness, efficiency, and satisfaction,” as the main outcome to evaluate Web-based health information tools [18]. However, usability is only 1 dimension of the user experience [19]. According to the technology acceptance model, technology acceptance and usage can be predicted by ease of use (ie, usability) and perceived usefulness [20,21]. Putting user experience in the context of Web-based health information tools, for which a patient is the end user, we therefore argue that we must evaluate not only *usability* but also *perceived usefulness* in terms of content and intention to use the tool. Where usability is related to the ease of use of a system, perceived usefulness addresses “the degree to which a person believes that using a particular system would enhance his or her job performance” [22]. The aim of this study is, therefore, to evaluate user experience (ie, *usability* and *perceived usefulness*) of Web-based health information tools among older patients. Important aspects of *usability* are the extent to which the tool meets the patients’ needs and abilities in terms of *navigation strategy* and *navigation problems* [23]. A Web-based health information tool high in *perceived usefulness* delivers its *content* in a way that satisfies the information needs of the user and increases their *intention* to use [24]. First, the *content* of Web-based health information tools should be considered in user experience evaluations for Web-based health information tools for older patients as the information needs of older patients might differ from those of younger patients [25]. Second, older patients who might be used to receiving health information through traditional media sources must perceive the Web-based information to be useful to develop an *intention* to actually use it.

As cancer is frequently a disease among older people [6], we will assess user experience with existing Web-based health information tools among older (≥ 65 years) cancer patients and survivors and their partners. We selected 7 Web-based health information tools with different functions (ie, information provision tools and preparatory tools). The results of this study can be used in the systematic development of Web-based health information tools for older cancer patients.

## Methods

### Study Design, Setting, and Sample

This study is part of a larger project in which we systematically developed a Web-based health information tool for older colorectal cancer patients. Participants were recruited from PanelCom, a panel of cancer patients who participated in previous studies with our research group and consented to be contacted again to participate in future studies. Participants were included if they were: (1) aged 65 years or older and (2) had been diagnosed with colorectal cancer or were a partner of a colorectal cancer patient or survivor. Approval for the study was obtained from the Institutional Review Board of the University of Amsterdam (2014-CW-64).

Think-aloud observations are a classic method to assess user experience of Web-based interfaces [26]. As older individuals might have short-term memory problems, valuable data might get lost when asking participants questions after using Web-based health information tools. The think-aloud methodology allows us to observe the actual reactions of the participants during the use of the tools. Another advantage is that the think-aloud method requires low numbers of participants. Throughout the literature, it has been found that only 5 to 9 participants can detect 80% to 90% of usability problems on a website [27-29]. However, the think-aloud method has also been criticized with respect to the validity of the self-reported data that it generates [30]. Previous research has therefore suggested combining think-aloud data with observational data [31]. Therefore, we recorded all sessions by video to be able to systematically observe how participants used the websites and preparatory tools. The think-aloud method enabled us to identify usability problems via observation and self-report. Moreover, the interview setting enabled us to query the participant concerning the perceived usefulness of the tools, specifically with regard to the content and intention to use the tool (see “Materials”). This combination of think-aloud data and interview data has been used previously to investigate usability and perceived usefulness [32].

### Materials

#### Cancer Information Websites

To identify characteristics of cancer information websites that best match the needs of the target group, we selected 3 existing websites that cancer patients might find when searching for information on the Internet, but the sources offering the information differed. When searching for Web-based health information, people commonly use general search engines such as Google, use short phrases or keywords, and tend not to look further than the first page of the search results [33]. We therefore selected a website that is the first hit on Google in the Netherlands when searching for the Dutch word for chemotherapy, which is 1 of the 3 most used treatments for cancer in the Netherlands [34]. This is a website with general information on chemotherapy that is owned by a pharmacist (website 1; [35]). As many Web-based health information consumers have difficulties in assessing the credibility of Web-based information [33], we next searched for a website from a seemingly reliable source, specifically a hospital. When searching for the Dutch words for “cancer” and “hospital,” the first hit on Google refers to a website for a specialized hospital for cancer patients in the Netherlands (The Antoni van Leeuwenhoek Hospital; website 2; [36]). As this study is part of a larger project in which we systematically developed a Web-based health information tool for older colorectal cancer patients, we selected the website of an expertise center for gastrointestinal cancer in the Netherlands (Gastrointestinal Oncology Cancer Center Amsterdam; website 3; [37]). Furthermore, we made sure in selecting the 3 websites that they differed from each other in terms of offering different modalities (ie, textual, visual, and audiovisual information) through which the information was presented and that they differed from each other with respect to various usability recommendations (eg, minimum 12-point font size, a button to increase text size, and static navigational elements), as proposed by Pernice and Nielsen [9]. Website 1 provided patients with textual information and used illustrations that clarified the text. The text on this website had a font size larger than 12 points but did not have the option to increase text size. The website did not have static navigational elements. Website 2 contained textual and audiovisual information. The text of this website had a smaller font size than 12 points and did not have the option to increase text size. The website did have static navigational elements. Website 3 contained textual and audiovisual information. The text on this website had a font size smaller than 12 points but had a button to increase text size. This website also had static navigational elements (eg, links and menus that do not change or move).

#### Question Prompt Lists

We used 3 Dutch Web-based QPLs for cancer patients. The first QPL was integrated into the website with chemotherapy information described previously (QPL 1; [38]). On this website, 4 QPLs were available concerning “preparation,” “a good conversation,” “side effects,” and “after the treatment.” The QPLs were in PDF format and were no longer than 1 page. The QPLs consisted of questions that patients might ask during consultations. Questions could be selected by ticking a checkbox in front of each question.

The second QPL was developed by the Dutch Breast Cancer Association for breast cancer patients and their family members (QPL 2; [39]). The homepage of the QPL contained 11 buttons that consisted of the main themes of the QPL and 3 other buttons for explanations and instructions, advice on preparing for consultations, and contact with an expert. The 11 main themes were further divided into 86 subthemes. The main themes were “diagnosis and treatment,” “questions for family members,” “hereditary and familial breast and ovarian cancer,” “breast cancer among older patients,” “breast cancer among younger patients,” “symptoms of breast cancer,” “work and re-integration,” “breast reconstruction,” “metastasized breast cancer,” “nutrition and exercise,” and “breast cancer among men.”

The third QPL was developed by researchers from the Academic Medical Center in Amsterdam for patients with esophageal cancer to prepare for their first consultation with the surgeon after surgery (as this tool was developed for research purposes and is not publicly available, we included a screenshot in Multimedia Appendix 1: QPL3). This QPL started with an explanation of the goal and content of the QPL and gave instructions to use the QPL. The QPL contained 76 questions across 9 themes: “operation and hospitalization,” “additional care,” “physical activity,” “social or emotional problems,” “nutrition,” “the probe,” “the future,” “physical assumptions,” and “medical care.” In addition, users could add their own questions.

#### Decision Aid: Values Clarification Tool

To the best of our knowledge, there was no publicly available Web-based decision support tool for cancer patients available at the start of our study. Therefore, we used a decision aid developed by researchers at the Leiden University Medical Center (this tool has been previously used for study purposes only; see Multimedia Appendix 2). This decision aid uses the values clarifications method, which aims to encourage the consideration of all relevant treatment options and/or attributes of options while lowering the processing burden so patients can adequately identify and integrate their values in forming a preference [40]. Values clarification methods can aid older patients to individually tailor treatment decision making according to their life values. The values clarification tool aimed to assess the relative importance of rectal cancer treatment outcomes. Patients were first asked to rate the importance of the occurrence of the best and worst probability of each possible treatment outcome (all else being equal) on a 4-point scale ranging from “not at all important” to “very important.” Next, patients were asked to rate the importance of 10 paired outcome scenarios on a 7-point scale, ranging from “a strong preference for scenario 1” to “a strong preference for scenario 2.” An example of a paired scenario was “Scenario 1: Fecal incontinence. Out of 100 people: 65 will have this, 35 will not. Sexual problems. Out of 100 people: 60 will have this, 40 will not. Scenario 2: Fecal incontinence. Out of 100 people: 50 will have this, 50 will not. Sexual problems. Out of 100 people: 70 will have this, 30 will not.” The questions in the values clarification tool were adaptive conjoint analysis based, meaning that the paired scenarios were tailored to each individual patient based on what they consider important tradeoffs [5].

### Procedure

Each participant evaluated the usability and perceived usefulness of 3 Web-based health information tools (ie, 1 of the 3 websites providing information, 1 of the 3 QPLs, and the values clarification tool; see Materials). Participants were first allocated to 1 of the 3 cancer information websites. We strove for an equal distribution of gender and being a cancer patient or survivor or a partner. All participants used the tools individually. Participants who were assigned to website 1 (ie, the website providing information on chemotherapy treatment for cancer) were also assigned to QPL 1, as this QPL was part of the same cancer information website. As QPL 2 was designed for female breast cancer patients, only female participants were assigned to this tool. As we had only 1 values clarification tool, all participants evaluated their user experience with this tool (see Table 1 for the distribution of participants across the tools).

We visited the participants at their homes. The sessions started with an explanation of the procedure, signing informed consent, and a short survey that assessed demographic information (ie, age, gender, and education), illness-related information (ie, diagnosis and treatment), and computer experience (ie, amount and purpose of computer use and usage of Web-based health information tools). Education was divided into low level of education (ie, primary education, lower vocational education, preparatory secondary vocational education, and intermediate secondary vocational education), middle level of education (ie, senior secondary vocational education and university preparatory vocational education), and high level of education (ie, higher vocational education and university). We provided all participants with the same hardware, using the same settings. Participants were instructed to perform several tasks according to the protocol. Participants were explicitly instructed to think aloud while executing tasks. It was emphasized that the goal of these tasks was not to test the quality of their computer skills but rather to test the usability of the Web-based health information tools. After finishing the tasks in the protocol, participants received a monetary reward of €20 for their participation.

To assess user experience, we developed an interview protocol containing different tasks (ie, search tasks, application tasks and evaluations; see Textbox 1). Some search tasks aimed to obtain insight in terms of the general navigation behavior of participants and contained the instruction to imagine a certain scenario and search for information one would like to receive in a particular situation. Other search tasks contained more elaborate instructions to search for specific information to assess information preferences. Evaluation tasks offered participants the opportunity to give their opinion about the content and usefulness for (parts of) the website or tool. Application tasks provided information about how participants use the website or preparatory tool. The protocol changed depending on the content of the Web-based health information tool visited for 1 search task (see Textbox 1; search task 7). The amount and types of tasks and questions remained the same.

**Description of questions in the observation protocol.**

1. Open the website (application task)

2. What is your first impression of this website? (evaluation)

3. Imagine you just got diagnosed with cancer. What information would you like to find on this website? Try to find that information (search task)

4. Were you able to find the information? Was it easy to find the information? What made it easy or difficult? Was the information understandable? (evaluation)

5. Go to the homepage (application task)

6. Was it easy to go back to the homepage? (evaluation)

7. Try to find (search task):

- information on how to prepare for a consultation with your health care provider (website 1)

- information on colorectal cancer (website 2)

- experiences of other patients on this website (website 3)

8. Were you able to find the information? Was it easy to find the information?

9. What made it easy or difficult? Was the information understandable? (evaluation)

10. Would you use this tool in the case that you were a patient for whom this tool is designed? (evaluation—intention to use)

### Analysis

All think-aloud observations were transcribed and coded independently by 2 researchers. Disagreements were resolved through discussion. We analyzed user experience on the basis of 2 dimensions: usability and perceived usefulness. Regarding the *usability*, the data from the think-aloud protocols were analyzed from 2 different perspectives as suggested by Van Waes [23]: (1) *navigation strategy* (ie, which navigation tools did the participant use?) and (2) *navigation problems* (ie, what were the navigation barriers the participant came across?). During the first round of coding, we initially used these 2 perspectives as coding categories.

All comments regarding usability could be classified under these codes, but as navigation strategy often led to navigation problems, we combined the 2 codes into 1 code: navigation strategy and problems. We subsequently identified 3 categories of navigation strategies and problems: (1) use of navigational elements, (2) layout, and (3) instructions. These 3 categories were used as subcodes during the second round of coding; all comments regarding usability could be classified under these subcodes.

Regarding the *perceived usefulness* of the Web-based health information tools, we coded whether participants had negative or positive remarks regarding the *content* presented on the website and whether participants had an *intention* to use the tools.

Regarding the negative and positive remarks regarding the content presented on the website, we identified 3 subcodes during the first round of coding: (1) satisfaction with information modality, (2) information preferences, and (3) satisfaction with comprehensibility. During the second round of coding, all positive and negative remarks regarding the content could be classified under these subcodes.

**Figure 1:**
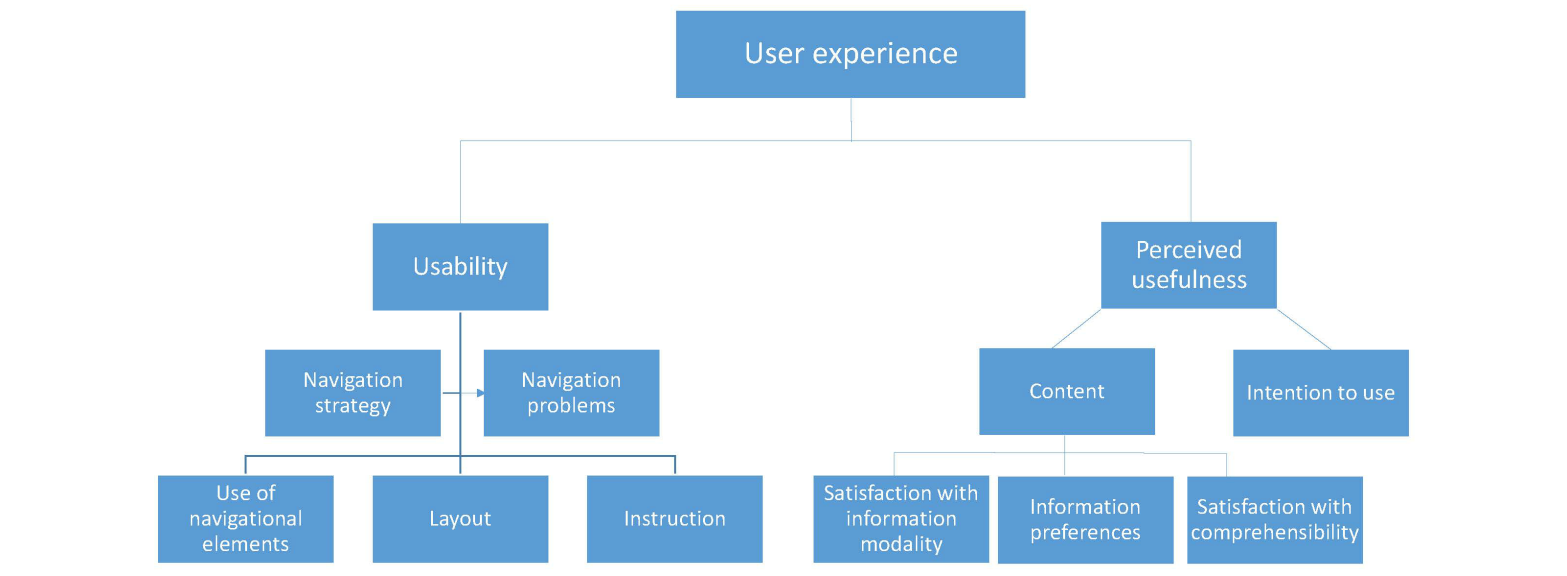
Code tree.

Regarding intention to use the tools, we coded whether and why participants indicated that they would or would not use the tool in the case they were a cancer patient or a partner of a cancer patient again. The codetree is shown in Figure 1.

## Results

### Participants and Their Characteristics

Participants were (colorectal) cancer survivors (diagnosed more than 2 years ago; n=12), colorectal cancer patients (diagnosed less than 2 years ago; n=3), and their partners (n=8). The median age of the participants was 73 (interquartile range 70-79). Participants’ characteristics are listed in Table 1.

**Table 1:** Participants’ characteristics.

		W1^a^ (n=6) n (%)	W2^b^ (n=8)	W3^c^ (n=9)	QPL 1^d^ (n=6)	QPL 2^e^ (n=7)	QPL 3^f^ (n=10)	Total or values clarification^g^ (n=23)
		n (%)	n (%)	n (%)	n (%)	n (%)	n (%)	n (%)
**Gender**								
	Male	4 (75)	2 (25)	5 (67)	4 (67)	0 (0)	7 (70)	11 (48)
	Female	2 (25)	6 (75)	4 (33)	2 (33)	7 (100)	3 (30)	12 (52)
**Age**								
	Median	74.5	76	70	74.5	73	73.5	73
	IQR^h^	73-77.5	72.25-81.25	67.5-79.5	73-77.5	72-82	67.5-79	70-79
**Education**								
	Low	2 (33)	3 (38)	4 (44)	2 (33)	3 (43)	4 (40)	9 (39)
	Middle	1 (17)	2 (25)	1 (11)	1 (17)	2 (29)	1 (10)	4 (17)
	High	3 (50)	2 (25)	4 (44)	3 (50)	2 (29)	4 (40)	9 (39)
	Other		1 (13)				1 (10)	1 (4)
**Diagnosis**								
	Colorectal cancer (patient)	2 (33)	1 (13)	0 (0)	2 (33)	0 (0)	1 (10)	3 (13)
	Colorectal cancer (survivor)	2 (33)	2 (25)	6 (67)	2 (33)	2 (29)	6 (60)	10 (44)
	Other cancer (patient)	0 (0)	0 (0)	1 (11)	0 (0)	0 (0)	2 (20)	2 (9)
	Other cancer (survivor)	1 (17)	1 (13)	0 (0)	1 (17)	0 (0)	0 (0)	1 (4)
	No diagnosis (ie, partners)	1 (17)	4 (50)	2 (22)	1 (17)	5 (71)	1 (10)	7 (30)
**Computer use per week^i^ (in hours)**								
	0-2	3 (50)	6 (67)	5 (63)	3 (50)	5 (71)	6 (60)	14 (61)
	2-10	1 (17)	1 (11)	0 (0)	1 (17)	0 (0)	1 (10)	3 (13)
	10+	2 (33)	2 (22)	3 (38)	2 (33)	2 (29)	3 (30)	6 (26)

^a^ Website 1: website about chemotherapy.

^b^ Website 2: website for Antoni van Leeuwenhoek Hospital.

^c^ Website 3: website for Gastrointestinal Oncology Center Amsterdam.

^d^ QPL 1: QPL on www.chemotherapie.nl.

^e^ QPL 2: QPL of the Dutch Breast Cancer Association.

^f^ QPL 3: developed by researchers of the Academic Medical Center in Amsterdam for patients with esophageal cancer.

^g^ Values clarification tool: a values clarification tool developed by researchers at the Leiden University Medical Centre.

^h^IQR: interquartile range.

^i^ Computer use: personal computer and/or tablet.

### Usability

#### Navigation Strategy and Problems

Although participants frequently commented that it was easy for them to find requested information (n=16; 70%), we observed that the large majority of the participants encountered problems in their navigation strategy and hence were not able to find the requested information (n=21; 91%). We identified 3 categories of navigation strategies that led to problems in optimally navigating the Web-based health information tools: (1) use of navigational elements, (2) layout, and (3) instructions.

#### Use of Navigational Elements

Participants often started to search for information in the center of a webpage (n=18; 78%) without paying attention to the structure of the website (ie, using a menu on the website to search for information). Website 3, for example, presented a large amount of information in the center of the webpage. The text contained several clickable links to other webpages. When we asked participants to search for specific information, most participants read the text in the center of the webpage and clicked on links provided in the text. They did not consider the menus at the top and on the left side of the webpage (n=8; 89%). Some participants commented that websites with 2 or more menu bars were too complex (n=5; 22%). Only 1 participant (4%) wanted to use the search bar to search for information but could not find the search bar.

For all Web-based health information tools, participants were required to scroll down to see an entire webpage. Overall, participants were able to and did not mind scrolling up and down (n=19; 83%), although 2 participants (9%) commented that the structure of the homepage would be easier to understand if they did not need to scroll.

QPL 2 presented a pop-up after the first question was selected. The pop-up presented the option to save the questions and to send the selected questions by email. However, this pop-up was confusing for some participants, as they had the feeling that the pop-up was an error message and that they did something wrong (n=2; 29%). Although there was an option to “continue anonymously,” some participants did not understand how to return to the QPL without saving the questions or leaving their email address (n=3; 43%).

When participants were given the task of returning to the homepage, they mostly used the arrow at the left top corner of the browser (n=12; 61%). Participants were not aware of the possibility of returning to the homepage of the website by clicking on the home button or on the logo of the website (n=17; 74%). Only website 1 had a “home” button to return to the homepage. However, this button was very small, and only 1 participant noticed it. One participant commented that they wanted to have a button with the text “back to the previous page” or “back to the homepage.” Website 3 had a button titled “back to care.” This button did not lead back to the previous page but to a completely different page on the website, which was confusing (n=5; 56%).

QPL 3 had 2 navigation possibilities. The first possibility was to go through all the questions in the QPL consecutively. The second option was to click on themes that were of interest to the participant and select the questions that were categorized under the specific theme and proceed by clicking on the next theme that was of interest. Participants mostly used the first option (n=9; 90%). Although participants went through the different themes and questions one by one, 2 (20%) did mention that they appreciated the subdivision into themes. The values clarification tool presented participants with one question at a time, which did not cause navigation difficulties.

#### Layout

Some participants were not able to read the text due to small font sizes (n=6; 26%) and/or a lack of contrast (n=5; 22%). Participants were not aware of the option to increase font size or were not able to find this option (n=2; 25%) that was presented by website 2. When we gave these participants instructions on how to increase the font size, they did appreciate this function.

Website 2 had a background consisting of a blurred illustration. Two participants commented that this was distracting because they did not know whether the illustration was blurred on purpose or whether this was due to their own visual decline (25%). The other 2 websites had plain backgrounds with colors that contrasted the text, which was greatly appreciated by the participants. Website 1 used different shades of brown colors. One participant (17%) mentioned that it was too difficult to see the differences, which made it difficult to read the text and to distinguish between buttons.

Website 1 and the values clarification tool presented buttons that were too small and too close together, resulting in participants clicking on the wrong button (n=7; 24%). Sometimes, participants were not aware of clicking the wrong button, leading to confusion as they saw a webpage with information that they did not expect or could not continue using the values clarification tool (n=5; 22%).

QPLs 1 and 3 used checkboxes that could be clicked on to select a question. QPL 2 used “+” and “-” symbols to select or deselect a question. These symbols were not always clear for participants (n=3; 43%). In addition, the same participants did not see that the question was added to their checklist after they clicked on it and when the “+” symbol changed into a “−” symbol. Furthermore, in the same QPL, the selected question changed from a black font into a gray font. Some participants did not notice this or were unable to see this change in colors (n=3; 43%).

The values clarification tool had a colored progress bar, which was appreciated by 2 participants (9%) but not noticed by the rest of the participants. One participant (4%) was color blind and could not see the progress in the bar.

#### Instructions

QPL 2 presented users with short instructions to help them with navigation while using the tool. Participants appreciated these instructions (n=2; 29%). One participant (14%) commented that they wanted instructions to navigate the website, for example, an instruction such as “click here if you want to have information on this topic.” QPL 3 started with an instruction on how to use and navigate through the tool. However, given that the instructions disappeared when participants were using the tool, some participants forgot these instructions (n=3; 30%). The instruction text was also considered too long, which resulted in some participants lacking the motivation to read the entire instruction (n=2; 20%). One participant commented that it would have been useful to receive smaller sections of the instructions while using the QPL (n=1; 10%).

### Perceived Usefulness

Perceived usefulness was measured in terms of satisfaction with the content of the Web-based health information tools and intention to use the Web-based health information tools. Regarding satisfaction with the content of the Web-based health information tools, we identified 3 categories: (1) satisfaction with information modality, (2) information preferences, and (3) satisfaction with information comprehensibility.

#### Satisfaction With Information Modality

Regarding the modality with which information was presented, the combination of text with a video was highly appreciated. Most participants commented that watching a video had added value after reading the text because it was difficult for them to process textual information only (n=11; 79%). Regarding illustrations, participants only found these useful when they clarified the text (n=6; 67%). One anatomical illustration on website 3 that used both Arabic and Latin numbers was difficult to understand. Illustrations that did not clarify the text, for example, a picture of a health care professional or a patient, received mixed comments. Some participants appreciated these illustrations (n=3; 13%), whereas other participants did not understand the reason why these illustrations were on the website and found these distracting (n=4; 17%).

#### Information Preferences

When we asked participants what information they would search for after having received a cancer diagnosis and/or starting a cancer treatment, they indicated a need for the following information: (1) information about cancer type and/or treatment (n=14; 61%), (2) personally relevant information, for example, information on a specific treatment they would receive (n=10; 43%), and (3) contact information for hospitals and health care providers (n=6; 26%).

Website 3 offered testimonials of patients’ experiences. Participants’ opinions about these testimonials differed greatly: most (n=7; 78%) highly appreciated this information, whereas some had no need for this information at all (n=2; 22%).

One participant (17%) mentioned that information about alternative treatment options was missing on website 1. According to this participant, a health care provider should give a patient the choice to undergo a treatment or not, and (s)he preferred to retrieve not only information about the recommended treatment but also about the alternatives.

There were some comments on the amounts of questions and themes in QPLs 2 and 3. Some participants indicated that there were too many questions or themes in these QPLs, which demotivated them to use the tool (n=4; 24%). One participant was overwhelmed by the amount of questions:

when I see all these questions, I think that there are so many things I should worry about.

#### Satisfaction With Comprehensibility

Despite extensive instructions and example questions presented before using the tool, most participants mentioned that they had difficulties understanding the questions in the values clarification tool (n=21; 91%). The illustration to visualize, for example, a 2 of 100 chance that the tumor would come back, was not clear to the participants (n=6; 26%). Furthermore, participants had difficulties understanding the questions in which 2 paired scenarios were offered (n=16; 70%; see Materials—Decision Aid: Values Clarification Tool—for an explanation on the paired scenarios). The instructions were followed by example questions, which aimed to help the user understand the types of questions. However, the fact that the example questions were not cancer related was considered confusing by some participants (n=3; 13%). Two participants (9%) commented that the text was easy to understand as no foreign languages or medical jargon was used.

The answer categories for the questions in which participants had to answer whether they had a preference for one scenario over the other were considered too ambiguous, as participants were asked to give their preference and to state how strong their preference was in 1 question (n=12; 52%). One participant commented that

it would have been easier to just answer whether one has a preference for one scenario over the other or whether one has no preference at all.

Another participant, while thinking aloud, said:

I will just answer that I have no preference, because I do not understand this question.

Other participants were also observed to answer with the “no preference” option (n=8; 35%).

Participants were bothered by the number and apparent similarity of questions (n=9; 39%). Two participants (9%) commented that it would take them too long to finish the tool and that it took too long before they came to the relevant questions. This is because the tool started with questions about each of the treatment consequences first, followed by questions on the combined consequences of the treatment. Concerning the instructions, these were perceived as containing too much text, although it was not clear for participants what they could expect. Participants mentioned that they would rather see the question while reading the instructions (n=7; 30%).

#### Intention to Use

Most participants mentioned that receiving information about their disease and treatment at home was very valuable, as it was very difficult for them to remember all the information presented during consultations (n=16; 70%). For example, 1 participant said that receiving information after the consultation is very useful as one can be too emotional to process information during the medical encounter. However, some participants mentioned that they would not use these types of websites as they expect to receive information from interpersonal communication with their health care providers and printed materials distributed by their health care providers. Another participant commented that they did not want any information at the time of diagnosis. However, the participant continued,

the added value of a website with information is that one can select the information that one needs at the moment one wants to have the information.

Participants had various needs regarding the amount of information. Some participants indicated that they were overwhelmed by large amounts of information (n=9; 39%), whereas other participants had a need for as detailed information as possible (n=4; 17%). One website offered the possibility of expanding the text for certain topics. This function was greatly appreciated by participants with both high- and low-information preferences (n=3; 33%).

Participants mentioned that the questions in the QPLs were useful for them and would help them to ask questions to their health care provider that they would not have thought of themselves (n=17; 74%). One participant, for example, mentioned that

you do not know what to expect before you have the consultation with your health care provider. It is very useful to see a list of possible topics that can be discussed.

Although most participants thought the QPLs were useful when preparing for consultation, 2 (9%) had doubts about actual usage during the consultation as they thought that the health care provider did not have time to answer all the questions. Two (9%) other participants considered preparing for a consultation by thinking of possible questions to be useful but would not use a Web-based tool for this as they are used to doing this by pen and paper. Some other participants commented that they would not use a QPL or would prepare questions for a consultation in another way, as they expect to receive the information they need from the health care provider (n=3; 13%).

Participants mentioned that they had difficulties understanding the aim of the values clarification tool. When the researcher explained the aim of the tool, some participants did mention that such a tool would be useful for them as they could understand that the topics in the values clarification tool were important to think about (n=11; 48%). However, 1 participant mentioned that this goal could have been achieved by asking just 1 question: “what is important in your life?” Another participant commented that the goal of the tool would also have been achieved simply by asking “what is most important for you: recurrence of the tumor or the side effects of the treatment?” Most participants would not use the tool themselves as they did not understand the questions (n=16; 70%). Another reason for not using the tool was because some participants preferred to discuss the issues presented in the values clarification tool with their health care provider rather than using a tool (n=7; 30%).

## Discussion

### Principal Findings

The aim of this study was to provide insight into the user experience with existing Web-based health information tools among older cancer patients. We evaluated 7 different Web-based health information tools in terms of usability (ie, navigation strategy and navigation problems) and perceived usefulness (ie, content evaluations and intention to use).

Regarding usability, we identified how older cancer patients navigate through a website and which navigation problems they encounter. Older cancer patients had difficulties navigating through websites that had complex structures (eg, multiple navigation bars). Moreover, some navigation problems were attributable to the layouts of the websites. For example, some buttons were too small to click on for older patients suffering from physical decline. In addition, the age-related problem of visual decline played a role in navigation problems due to layout in that older patients had difficulties distinguishing colors that had low levels of contrast. Regarding the content that was presented on the websites, we found that older patients appreciated it when information was presented in different modalities (ie, text combined with illustrations or video). However, this combination was mostly appreciated if it was used to clarify the text and less for aesthetic reasons. Next, we found that older cancer patients and their partners varied greatly in terms of the amount of information they wanted to receive. Some patients wanted to receive as much information as possible, whereas other patients wanted to receive less information or no information at all. This finding is consistent with literature that found that older patients do not always want complete information on their disease [25]. All patients appreciated a website for which there was a possibility to expand information so that they could select the information they wanted to receive themselves.

The great effort it took for older adults to digest large amounts of information is probably also the reason why they preferred to only read what is applicable to their own situation, without having to filter it from among general information. This is in line with previous research that suggests that people find it increasingly difficult to concentrate on relevant information as they get older [41] and that older patients read large amounts of text when available [42].

Regarding the perceived usefulness of the Web-based health information tools, older adults overall indicated willingness to use both the health information websites and the preparatory tools. Reasons for not using the tools were that they would rather receive or discuss the information with a health care provider, that they preferred to receive offline information, or that they did not understand the content, which was the case for the values clarification tool. Similar results were found in usability testing of a comparable DA for chronic obstructive pulmonary disease patients [43].

### Strengths and Limitations

Although our participants were cancer patients or survivors and their partners, we asked participants to project themselves into the hypothetical situation that they were just diagnosed with cancer or just about to receive a treatment. The use of such so-called “analog patients” is documented in meta-analysis as a valid method [44]. However, it may be more difficult even for cancer survivors to imagine the perceived usefulness of the system to a person newly diagnosed with cancer. To illustrate, not all information that was presented on the websites that we selected was applicable to the situations of the patients and their partners, which might have resulted in information that was not personally relevant. This possibly resulted in participants that were not as committed as the intended users of the websites and tools would be. Furthermore, although the usability problems of newly diagnosed patients might be similar to those of our analog patients, newly diagnosed patients may be more upset by usability problems that would make them unsure of whether the information they found applied to them or whether the decision they reached with the aid of the tool was the right one. This might affect the interpretation of the results and indicates that we must take even small usability problems very seriously. Moreover, prototypes of newly developed Web-based health information tools for older people should also be tested among recently diagnosed patients and partners.

We observed a difference between self-reported data and our observational data regarding the self-reported ease of finding information and the observed difficulty with actually finding the requested information, which points to the importance of using both self-reported data and observational data in user experience research. A possible explanation for this discrepancy is that participants may have given a socially desirable answer as the researcher was sitting next to the participant, although the researcher explained beforehand that the goal of the study was not to test the skills of the participant but the usability of the website.

### Comparison With Prior Work and Practical Implications

Previous guidelines focused on usability aspects of Web-based health information tools for older people, whereas this study also provides insights into perceived usefulness. Regarding usability, our study confirms some of the existing recommendations, refutes others, and suggests recommendations that are not mentioned in the existing guidelines. As Internet experience is increasing rapidly among older adults, some prior recommendations are no longer applicable to the current generation of older people. For example, our study showed that older website users can easily navigate through a pull-down menu—a nonstatic navigational element, whereas Pernice and Nielsen [9] more than a decade ago found that older Internet users had difficulties using these. The same authors [9] advise against scrolling down in a webpage. However, this study shows that most older users have no problems in doing so anymore.

The findings of this study confirm other existing recommendations. First, participants still had difficulties in reading small font sizes. It is important that websites designed for older users have adequate font sizes by default as participants were not able to find the button to increase font size. Second, similar to what we found in our study, Pernice and Nielsen [9] described that older users clicked on the back button in the browser to return to the homepage. Older users in our study were also not aware that clicking on the company logo would lead them back to the homepage. The recommendation to add a link called “Home” on all website pages except on the homepage and preferably in the horizontal navigation bar is therefore still applicable. Third, Pernice and Nielsen [9] recommended leaving space between links and to make the immediate area surrounding the link part of the link as older users have more difficulties with accurately clicking on small targets. This result is confirmed in our study, in which we found older users to be confused when they clicked on the wrong link or button or when nothing happened after misclicking the link.

Pernice and Nielsen [9] recommend presenting informational messages, including error messages, clearly and in a nonthreatening way. Although error messages were not common on the websites we tested in this study, we noticed that older users react in a confused or anxious manner when a pop-up unexpectedly shows up. Even when the pop-up is not an error message, participants interpreted it as such, which made them anxious. Another recommendation that was not found in the existing guidelines but that we would like to add is based on our finding that older users focus on the main text on a website instead of on navigational elements such as navigation bars. We therefore recommend presenting navigational elements in the center of the homepage, which will help older users immediately make a navigational choice without being distracted by possible irrelevant information. We also recommend avoiding large amounts of main text on the homepage and to display options on one page. For instance, if a clear overview with options is provided first, users can make a conscious choice regarding which information they want to read and click on the link or button with information that is relevant to them. To satisfy both users who prefer detailed information and users who want to read only key information, give text the ability to “pull out” for users who want to read more detailed information. This was highly appreciated by both groups on website 2. Make sure to use static menus and to not use more than 1 layer for pull-out menus to avoid users getting lost in the website.

Finally, this study builds on the existing guidelines in terms of providing insights regarding how to incorporate preparatory tools such as QPLs and values clarification tools. Based on the results of the think-aloud observations, we recommend providing clear instructions on how these tools can be used that are also available when using the tool. It is also recommended to limit the number of questions and themes in QPLs to a maximum of 20 predefined questions per QPL, to make 1 question visible at a time and to provide the possibility of adding additional personal questions. To be able to provide the user with a personal overview of all selected questions in order of priority, the option to add or to not add a selected question to this personal list (QPL) or answer (values clarification tool) should be provided, as well as the ability to prioritize the importance of questions, for example, by asking the user to indicate whether the topic or question is “not important (0)” to discuss, “rather important (1),” “important (2),” or “extremely important (3).” The ability to store this personal list, to print it out, or to email it should be incorporated into the tool. Textbox 2 gives a summary of recommendations that can be derived from this study and previous studies.

One of the benefits of Web-based health information tools compared with traditional health information sources (eg, printed patient leaflets) is that information can be tailored to meet the individual preferences of individual patients [45]. Moreover, tailored information has been found to be processed more deeply, contain less redundant information, and is perceived more positively by users [46]. Although these results were found in a different context and in a younger sample, we expect that these benefits could also apply to older cancer patients as our results confirm that older cancer patients vary greatly in terms of their information preferences (ie, the amount and type of information they want to receive), literally evidenced by comments regarding the need to receive information that is personally relevant. Tailoring information according to patients’ preferences would therefore make information more personally relevant, allow deeper information processing [47], and would contain less redundant information, which could be particularly beneficial to older patients considering age-related cognitive limitations [48].

Although the text of the values clarification tool was often perceived as too difficult to understand, participants thought that the goal of the values clarification tool (ie, a tool that would support them in thinking about which treatment consequences are most important for them) would be very useful. This is in line with a study [49] in which it was found that patients perceive such a values clarification tool as useful. A Web-based values clarification tool should therefore offer text or questions that are easy to understand and that prompt them to start thinking about their preferences. For example, a QPL consisting of 3 simple questions (ie, “what are my options?,” “what are the possible benefits and harms of those options?,” and “how likely are the benefits and harms of each option to occur?”) has been designed [50]. The authors found that health care providers took patient preferences concerning treatment options into consideration after patients asked these 3 questions.

All Web-based health information tools were easier to use for older patients when they were provided with short instructions during use. Instructions that were given before Web-based health information tools were not remembered, if read at all. Short instructions should be provided while using the tool and should only apply to the specific function that is used at that time.

**Recommendations for the development of Web-based health information tools for older patients.**

**General guidelines**

Recommendations based on current think-aloud observations:

- older people often use tablets: it is important that the site is suitable for a tablet;
- provide the ability to print information or to save as a PDF or send to email.

Recommendation based on both the current think-aloud observations and literature:

login must be simple.

Recommendation based on prior guidelines or literature:

ensure that the home page loads quickly.

**Access to information (structure and navigation)**

Recommendation based on current think-aloud observations:

avoid large amounts of information on a page. If possible, display options on 1 page, for example, first provide an overview with options, and then (after visitors choose what information they wish to read) the relevant information.

Recommendations based on both the current think-aloud observations and literature:

the design must focus on easy-to-use navigation tools:

- show a navigation bar on every page on the same place. (top)
- use a prominent homepage button on each page.

hyperlinks must be distinguishable from other text and easy to click on (eg, not too close to other text). Change the color if a link is clicked.

Recommendation based on prior guidelines or literature:

make sure that links go directly to the content.

**Information (text, illustrations, video, multimedia)**

Recommendations based on current think-aloud observations:

- allow text to “pull out” for users who want to read more detailed information (see eg,: http://www.avl.nl/behandelingen/chirurgie-bij-dikke-darmkanker/ under “What is going to happen”);
- provide a clear explanation of illustrations: what exactly is there to see?

Recommendations based on both the current think-aloud observations and literature:

- combine strategies (audiovisual and text) so that older people have a choice. This is important because older people often want to control the pace at which they obtain information (which may be less possible with audiovisual information);
- a combination of personalization and audiovisual information enhances information memorization;
- adding images to a website improves satisfaction with the attractiveness of a website. Illustrations that explain the text are considered most useful. The images need to be carefully tested in the target group;
- large, readable font (so that a button to enlarge text is not required);
- text in a contrasting color background;
- write for users; do not use difficult language or technical terms;

Recommendations based on prior guidelines or literature:

- a website with a personal video (information provided by a patient) and text can increase satisfaction with the website;
- terms such as senior, older, or age-related terms can be used on the site. However, do not use stereotypes or patronizing text.

**Usability**

Recommendation based on both the current think-aloud observations and literature:

a search function is rarely used by the elderly. If this function is installed:

- make the search field very clear (eg, put the word “Search” clearly in front of open fields where users can search);
- the search engine should be easy to use and also work when punctuation is used;
- always repeat the search terms clearly above the search results;
- make sure the results are visible on the page without scrolling.

- buttons and other interactive objects must be easily clickable;
- error messages should be understandable and visible;
- when a pop-up is used, ensure that all information fits into the screen so that users do not have to scroll.

Recommendation based on prior guidelines or literature:

use static menus (no pull-down menus or other moving elements).

**If something needs to be filled in a form on the website:**

Recommendation based on prior guidelines or literature:

if you are asked for a date, use this format:

- select month by means of a drop-down list of months in chronological order;
- type date into an empty field;
- type year in 4-digit format in an empty field.

- if there are errors in a form, accept all the correct information and show users only the fields that need to be changed. Explain what the user should do to correct the error at the top of the form;
- ask users not to enter a salutation. Use a drop-down list if this information is required.

**Preparatory tools (QPLs or values clarification tools)**

Recommendations based on current think-aloud observations:

- provide clear instructions, which are also available when using the tool;
- limit the number of questions and themes in QPLs (up to 20 questions per topic);
- make 1 question at a time visible with the ability to add or not add the question to a personal list (QPL) or answer (values clarification tool);
- give an overview of all selected questions in order of priority and the option to add additional (personal) questions;
- provide the ability to store the list, print it out, or email it.

### Directions for Future Research

In addition to using existing guidelines for website development for older adults in general, our study shows the importance of taking the specific target group, in this case, older cancer patients, into consideration, as this group differs from a more general older target group. Future studies should investigate the user experience of other older patient groups as patients with other diseases might have different information needs or Web-based health information tools might have other functions such as medication reminders for patients with chronic diseases. Next, as the Netherlands is one of the countries with the highest Internet access among adults aged 65 years and older [51], future research is needed in countries where there are lower levels of Internet access.

Previous research concluded that QPLs can improve communication and psychological and cognitive outcomes in cancer patients (see [4] for a systematic review of the literature), and this was also found for older cancer patients [52]. This suggests that QPLs are useful tools to be developed and implemented for various diagnostic tests and treatments in cancer care. Although most participants considered a QPL to be a highly useful tool, this was not true for every older cancer patient. We identified certain reasons why older cancer patients would not intend to use a (Web-based) QPL, such as a preference for paper and pen and relying solely on interpersonal communication during consultation. The impression that the health care provider would not have time to answer the questions was also mentioned as a barrier, which is in line with previous research identifying barriers that patients have when discussing certain topics during consultations [53]. We therefore recommend that QPLs should not contain a large number of questions and should prioritize questions so that patients can ask their most important questions first, without increasing the consultation time (see Practical Implications). Future research should further investigate barriers for using Web-based health information tools such as QPLs.

The values clarification tool was also designed to be used to prepare patients for their consultation with their health care provider and to support the conversation about the weighing of benefits and harms of treatment. Participants indicated that the number of questions used in the values clarification tool was too extensive and that the importance of the outcomes could have been assessed by asking them in one direct question. However, the purpose of the adaptive conjoint analysis is that the relative values are assessed, that is, the importance of an outcome in relation to the other outcomes. Participant comments indicated that they would rather discuss the advantages and disadvantages of a treatment with their health care provider instead of using the tool, which might differ when participants actually used the tool in combination with interpersonal communications with their health care provider. As the values clarification tool is designed and had been used in the context of a clinical study only in which the tool was combined with consultations with health care providers, the comments of the participants in this think-aloud study were that they would rather discuss these benefits and harms of treatment with their health care provider are therefore not unexpected. A recent literature review on the effectiveness of decision aids for older adults indicated that patient outcomes seemed to be better when participants received the decision aid from their clinician during the consultation than when it was delivered by a researcher before the consultation [54,55]. This suggests that decision aids might be particularly useful for older adults when successfully integrated with interpersonal communication during the consultation. However, only 2 studies in which the decision aid was delivered during the consultation were included in the review [56]. The same might hold true for the QPLs. Future studies should therefore examine the added value of these tools when offered by the health care provider during the consultation.

### Conclusions

This study shows how older cancer patients use and evaluate Web-based health information tools. Older cancer patients are fully able to use Web-based health information tools and perceive these tools as highly useful in their search for health information and to prepare for interpersonal communication with their health care providers. However, older patients experienced navigational problems that can hinder optimal user experience with these tools. This study unmasked these navigation problems along with specific user preferences. We used our results to propose improvements for the design of Web-based health information tools for optimal user experience among older patients.

## Appendix

Multimedia Appendix 1 Screenshot of QPL 1.

Multimedia Appendix 2 Screenshots of values clarification tool.

## Abbreviations

QPL: question prompt list
