# Supplementary material for: Older Cancer Patients’ User Experiences With Web-Based Health Information Tools: A Think-Aloud Study
Source: J Med Internet Res. 2016 Jul 25;18(7):e208. doi: 10.2196/jmir.5618 (PMC4977420; doi:10.2196/jmir.5618)

Multimedia Appendix 1

Screenshot QPL 1: developed by researchers of the Academic Medical Center in Amsterdam for patients with esophageal cancer. The blue bars contain the different themes in the QPL. When the bars are expanded the questions belonging to that theme become visible.

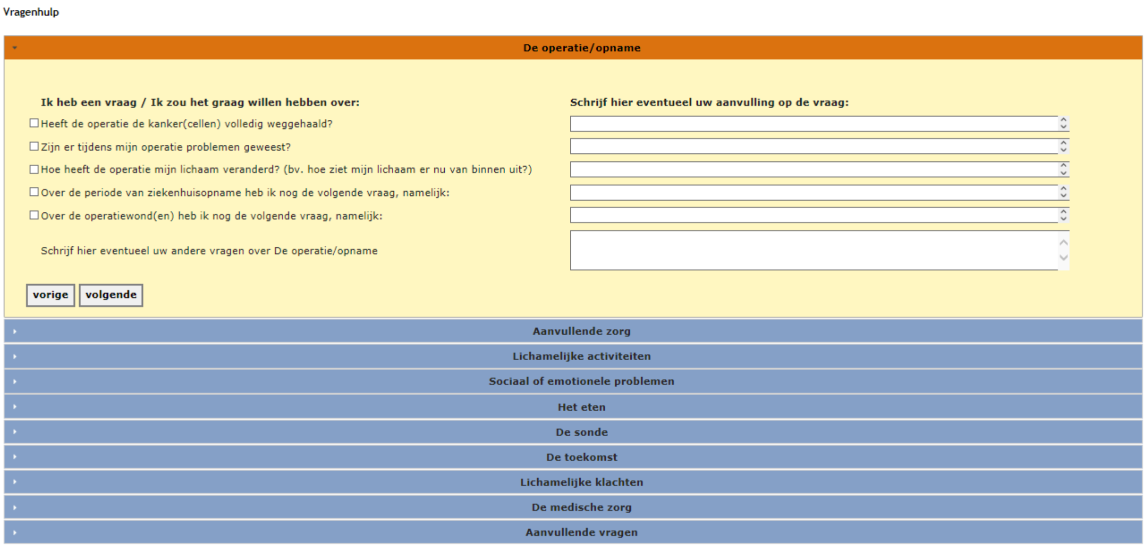

Supplement: Multimedia Appendix 1 [file jmir_v18i7e208_app1.pdf]
