# Supplementary figures and images for: Older Cancer Patients’ User Experiences With Web-Based Health Information Tools: A Think-Aloud Study
Source: J Med Internet Res. 2016 Jul 25;18(7):e208. doi: 10.2196/jmir.5618 (PMC4977420; doi:10.2196/jmir.5618)

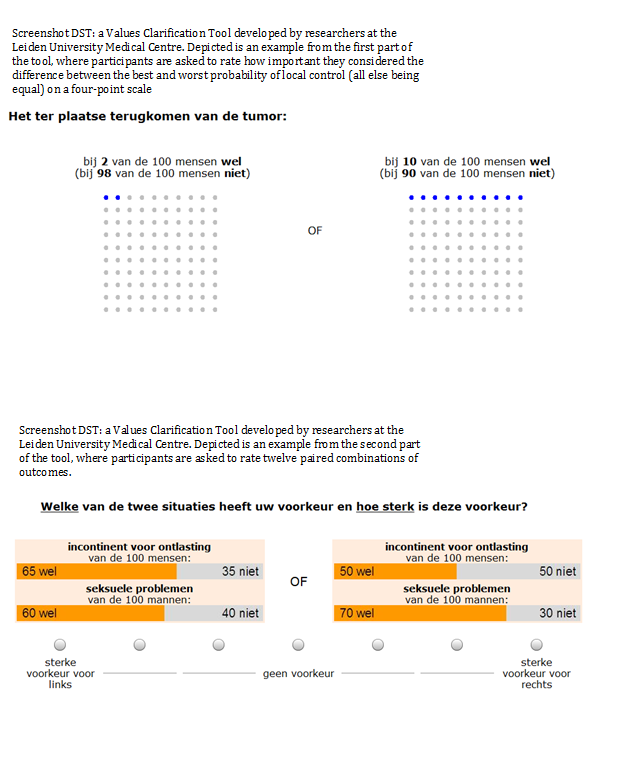

Supplement: Multimedia Appendix 2 [file jmir_v18i7e208_app2.png]
